# Supplementary material for: Augmenting biologging with supervised machine learning to study in situ behavior of the medusa Chrysaora fuscescens
Source: J Exp Biol. 2019 Aug 23;222(16):jeb207654. doi: 10.1242/jeb.207654 (PMC6739807; doi:10.1242/jeb.207654)
Supplement: Supplementary information [file jexbio-222-207654-s1.pdf]

Table S1. Summary of laboratory and *in situ* deployments of ITAG on *Chrysaora fuscescens*

Laboratory	Animal ID	Date deployed	Tag ID	Tag data (sec)	Video footage (sec)	Drogue depth (m)	Location collected	Date collected
	T1-1	18/05/18	e2	2321	1916	N/A	36.7968, -121.8298	18/05/18
	T2-1	18/05/21	b7	4562	2866	N/A	36.7968, -121.8298	18/05/18
	T2-2	18/05/21	e2	3110	3004	N/A	36.7968, -121.8298	18/05/18
	T3-1	18/05/31	b7	4257	4044	N/A	36.86749, -121.9027	18/04/06
In situ							Location deployed	Time deployed (PDT)
	S1-1	18/04/24	24	23479	264	5	36.8315 -121.8767	10:42
	S1-2	18/04/24	3c	2166	614	None	36.8355, -121.8750	12:06
	S1-3	18/04/24	e2	20571	789	9	36.8383, -121.8759	12:40
	S2-1	18/05/14	24	16893	1619	9	36.8243, -121.9247	11:00
	S2-2	18/05/14	e2	19464	1374	9	36.8219, -121.9234	12:21
	S3-1	18/05/17	e2	10289	1332	9	36.8397, -121.8854	12:42

Journal of Experimental Biology • Supplementary information

	S3-2	18/05/17	24	2484	0	9	36.8333, -121.8827	13:52
	S3-3	18/05/17	b7	36400	975	9	36.8302, -121.8790	14:21

Footnotes: During the S3-2 deployment, the ROV lost track of the jellyfish almost immediately after release due to strong currents and no viable footage of behavior was recorded. In four out of the eight deployments (S1-1, S1-3, S3-1, and S3-2), the tag was still attached to the jellyfish at the time of retrieval. In the remaining four deployments (S1-2, S2-1, S2-2, and S3-3), the jellyfish was no longer attached.

Table S2. Summary of laboratory and *in situ* video footage annotations

Laboratory	Animal ID	Total annotated footage (sec)	Activity			Tether influence			Unannotated tag data after footage (min)
			Drift (sec)	Swim (sec)	Unknown (sec)	Taut tether (sec)	Slack tether (sec)	Unknown (sec)	
	T1-1	1916	0	1916 (100%)	0	1093 (57.0%)	816 (42.6%)	7 (0.4%)	N/A
	T2-1	2866	55 (1.9%)	2811 (98.1%)	0	575 (20.1%)	2253 (78.6%)	38 (1.3%)	N/A
	T2-2	3004	4 (0.1%)	3000 (99.9%)	0	550 (18.3%)	2454 (81.7%)	0	N/A
	T3-1	4044	311 (7.7%)	3733 (92.3%)	0	0	4044 (100%)	0	N/A
Total laboratory		11830	370 (3.1%)	11460 (96.9%)	0	2218 (18.7%)	9567 (80.9%)	45 (0.4%)	N/A
In situ	S1-1	154	0	154 (100%)	0	24 (15.6%)	102 (66.2%)	28 (18.2%)	388
	S1-2	590	3 (0.5%)	350 (59.3%)	237 (40.2%)	25 (4.2%)	136 (23.1%)	429 (72.7%)	37
	S1-3	653	5 (0.8%)	631 (96.6%)	17 (2.6%)	127 (19.4%)	207 (31.7%)	319 (48.9%)	339
	S2-1	1431	7 (0.5%)	1415 (98.9%)	9 (0.6%)	0	727 (50.8%)	704 (49.2%)	262

Journal of Experimental Biology • Supplementary information

	S2-2	1347	0	1347 (100%)	0	0	1285 (95.4%)	62 (4.6%)	311
	S3-1	1158	31 (2.7%)	1116 (96.4%)	11 (0.9%)	69 (6.0%)	285 (24.6%)	804 (69.4%)	146
	S3-2	0	N/A	N/A	N/A	N/A	N/A	N/A	54
	S3-3	762	33 (4.3%)	721 (94.6%)	8 (1.0%)	80 (10.5%)	83 (10.9%)	599 (78.6%)	590
Total *in situ*		6095	79 (1.3%)	5734 (94.1%)	282 (4.6%)	325 (5.3%)	2825 (46.3%)	2945 (48.3%)	2127

Table S3. Tether-influence and activity predictions for individual jellyfish

Deployment ID	Representative pulse frequency (pulses/sec)	Unannotated data* classified as influenced	Unannotated data* classified as drifting (out of time classified as uninfluenced)
S1-1	0.260	35.1%	19.1%
S1-2	0.487	21.2%	0%
S1-3	0.615	6.0%	0.9%
S2-1	0.520	3.3%	0%
S2-2	0.466	5.0%	0.1%
S3-1	0.275	28.2%	5.6%
S3-2	0.422	9.8%	0.6%
S3-3	0.380	11.4%	2.7%

* Rightmost column of Table S2.

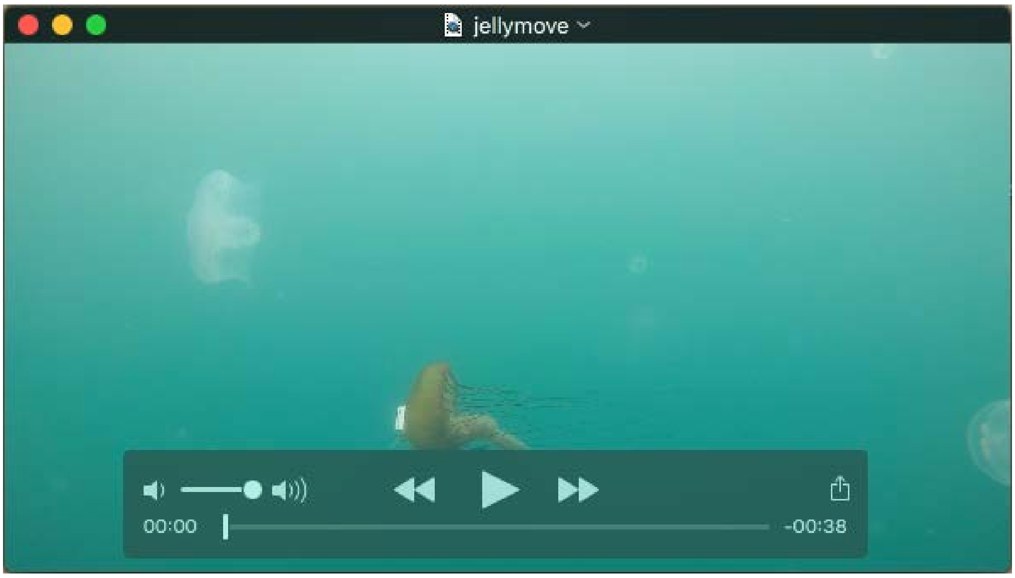

Movie 1. Examples of annotated *in situ* and laboratory footage. In order, uninfluenced *in situ* swimming, tether-influenced *in situ* swimming, *in situ* swimming with unknown tether status, uninfluenced *in situ* drifting, tether-influenced *in situ* drifting, and swimming and drifting in the MBARI Test Tank.

Journal of Experimental Biology • Supplementary information
